# A CD8^+^ T Cell Infiltration–Driven Prognostic Signature for Gastric Cancer: Bridging Tumor Immunity and Clinical Outcomes

**DOI:** 10.1155/ijog/6629479

**Published:** 2025-06-13

**Authors:** Yiting Qian, Bo Sun, Linying Lai, Fengying Xu, Ruilin Liu, Wenzhuo Yang

**Affiliations:** ^1^Department of Gastroenterology and Hepatology, Digestive Disease Institute, Tongji Hospital, Tongji University School of Medicine, Shanghai, China; ^2^Animal Experiment Center of Tongji Hospital, Tongji University School of Medicine, Shanghai, China; ^3^Department of Pulmonary and Critical Care Medicine, Tongji Hospital, Tongji University School of Medicine, Shanghai, China

**Keywords:** CD8^+^ T cell heterogeneity, gastric cancer, immunotherapy targets, prognostic biomarkers, single-cell RNA sequencing, tumor microenvironment

## Abstract

**Background:** CD8^+^ T cells play pivotal roles in antitumor immunity, where infiltration levels often correlate with favorable prognosis. However, the functional heterogeneity of CD8^+^ T cell subsets within the gastric cancer (GC) tumor microenvironment (TME)—particularly their divergent impacts on tumor progression, immunotherapy response, and clinical outcomes—remains poorly characterized.

**Methods:** We integrated single-cell RNA sequencing (scRNA-seq) data from 23 GC tissues (GEO: GSE150290) with bulk transcriptomic profiles from TCGA-STAD to dissect CD8^+^ T cell heterogeneity. Analytical pipelines included unsupervised clustering, pseudotime trajectory analysis, and protein–protein interaction (PPI) network construction to identify survival-associated hub genes. Differential gene expression, functional enrichment, and experimental validation were performed to confirm clinical relevance.

**Results:** scRNA-seq resolved CD8^+^ T cells into five functionally distinct subsets: naïve/memory, exhausted, and three cytotoxic subpopulations. Among these, cytotoxic CD8^+^ T1 cells exhibited the strongest prognostic relevance, with high infiltration correlating to improved survival and enrichment in G2-grade tumors. Pseudotime analysis revealed differentiation trajectories from naïve to exhausted subsets, accompanied by metabolic and immune checkpoint pathway alterations. PPI network analysis identified SELL, CD79B, and RAMP2 as hub genes, all significantly linked to survival and differentially expressed across tumor grades/stages. Experimental validation confirmed that SELL, CD79B, and RAMP2 knockdown suppressed GC cell proliferation, underscoring their functional roles.

**Conclusion:** Our study unveils the landscape of CD8^+^ T cell heterogeneity in GC and proposes a three-gene signature (SELL/CD79B/RAMP2) with dual prognostic and therapeutic potential. These findings provide actionable insights for stratifying patients, tailoring immunotherapy regimens, and developing novel targets to enhance antitumor immunity in GC.

## 1. Introduction

Gastric cancer (GC) ranks as the fifth most prevalent malignancy and the fourth leading cause of cancer-related mortality globally [1], with over 70% of patients diagnosed at advanced stages where therapeutic options yield limited survival benefits [2, 3]. While immunotherapy has revolutionized oncology—notably in melanoma and hematologic malignancies—its efficacy in solid tumors like GC remains suboptimal [4]. This disparity stems from the immunosuppressive tumor microenvironment (TME), characterized by hypoxia, nutrient deprivation, and dysfunctional immune cell states, which collectively impair cytotoxic T cell activity [5–9].

CD8^+^ T cells, pivotal mediators of antitumor immunity, exhibit functional heterogeneity within the TME. However, their subset-specific roles in GC progression, therapy resistance, and immune checkpoint regulation remain poorly defined. Recent advances in single-cell RNA sequencing (scRNA-seq) have enabled high-resolution dissection of TME complexity, revealing cell type–specific signatures linked to tumor evolution and immunotherapy response [10–14]. For instance, scRNA-seq studies in liver and lung cancers have uncovered T cell exhaustion trajectories and activation-impaired subsets predictive of clinical outcomes [15–18]. Yet, a systematic characterization of CD8^+^ T cell heterogeneity in GC—critical for developing targeted immunotherapies—is lacking.

Here, we integrate scRNA-seq of 23 GC tissues with bulk transcriptomic data to resolve CD8^+^ T cell heterogeneity. We delineate subset-specific functional states, differentiation trajectories, and immune checkpoint dynamics. Crucially, through in vitro functional validation, we demonstrate that three hub genes (SELL, CD79B, RAMP2)—identified via protein–protein interaction (PPI) networks—directly regulate GC cell proliferation. These findings not only map the landscape of immune dysfunction in GC but also provide actionable biomarkers for personalized immunotherapy regimens.

## 2. Materials and Methods

### 2.1. scRNA-seq and RNA-seq Data

GC scRNA-seq was downloaded from the Gene Expression Omnibus (GEO) database (GSE150290). And 123,302 cells from 23 GC tissue samples were screened. From the TCGA Xena database (https://xenabrowser.net/datapages/), the transcriptome and sample phenotypic data of TCGA-STAD were downloaded; a total of 408 samples were obtained, of which 377 were tumor tissues (27 of the samples had no survival information and were excluded from subsequent analysis) and 31 were normal tissues. The fpkm expressed values were used for subsequent analysis. A total of 69 KEGG metabolic gene sets were obtained from c2.cp.kegg.v2023.1.Hs.symbols.gmt from MSigDB. The 50 hallmark gene sets (h.all.v2023.1.Hs.symbols.gmt) were obtained from MSigDB, representing well-defined biological processes and states relevant to cancer immunology.

### 2.2. CD8^+^ T Cell Subsets' Identification

We used the Seurat R package to map the expression profile of CD8A and CD8B (the classic CD8^+^ T cell markers) and determined the CD8^+^ T cell population. Then, UMAP cluster analysis was performed, and each cell subset was annotated according to the expression distribution of CD8^+^ T cell subtypes. Then, we counted the proportion of each subset of CD8^+^ T cells and used the ggplot2 R package to draw a bar chart. Hypervariable genes of each subtype were examined by using the Seurat R package. The top 10 highly variable genes of each subtype were selected, and the heatmap R package was used to draw the heatmap of gene expression. We annotated marker genes from previously reported studies [19].

### 2.3. Cellular Composition of CD8^+^ T Cell Subset

Seurat R package was used to obtain the hypervariable genes of each subset of CD8^+^ T cells, which were screened by the Bonferroni correction *p* value < 0.05. For screening of gene expression, we used CIBERSORTx (https://cibersortx.stanford.edu/) tools and got CD8^+^ T cell subtype of GC signature matrix file. The transcriptome data of TCGA-STAD were collated, and the expressed count value was converted into CPM as the input file of CIBERSORTx to estimate the content of CD8^+^ T cell subtypes in GC (Supporting Information 1). Then, we used the surv_cutpoint function from the survminer R package to determine optimal cutoffs for cellular composition analysis. Using maximally selected rank statistics, TCGA-STAD samples were divided into high- and low-level groups. And we used the Survival package and survminer package to plot the survival curve of KM (Supporting Information 2). The phenotypic data of TCGA-STAD were sorted. Boxplots were drawn to show the difference in cellular composition between gender, grade, stage, age, diagnosis, disease types, and tissues.

### 2.4. Cell Fraction Estimation

To infer each subset of CD8^+^ T cell infiltration levels in TCGA-STAD, we used the expression matrix of the three subtype CD8^+^ T cells as a signature matrix, TCGA-STAD log2 transformed CPM matrix as a mixture, and CIBERSORTx tool to estimate the infiltration level for each primary tumor sample.

### 2.5. GO and KEGG Enrichment Analysis

To identify gene sets or pathways relatively enriched in the cytotoxic CD8^+^ T Cell Cluster 1 (cytotoxic_CD8_T_cell_1), we performed Gene Ontology (GO) and Kyoto Encyclopedia of Genes and Genomes (KEGG) enrichment analyses. First, differentially expressed genes (DEGs) were identified using the FindMarkers function in the Seurat package, with a threshold of log2 fold change > 0.5 and an adjusted *p* value < 0.05. All other parameters were retained at their default settings.

### 2.6. HALLMARK Gene Set Enrichment Analysis

To explore which pathway is enriched in cytotoxic_CD8_T_cell_1, we first sort genes by log2 fold change and then use hallmark genes and the GSEA function from clusterProfiler to perform GSEA enrichment analysis. The enrichment result was visualized with a heatmap.

### 2.7. Survival Analysis

To explore whether the enrichment level of each CD8^+^ T cell correlated with survival, we first grouped tumor samples from TCGA-STAD into high and low groups for each subtype with the surv_cutpoint and surv_categorize functions from the survminer package based on the infiltration levels returned by CIBERSORTx. Then, we performed survival analysis between high and low groups with the survival package.

To define these groups, the surv_cutpoint function from the survminer R package was used to find the optimal cutpoint for cytotoxic CD8^+^ T Cell 1, cytotoxic CD8^+^ T Cell 3, and naïve memory CD8^+^ T cell infiltration score. This function uses maximally selected rank statistics, which assume that an unknown cutoff in the independent variable *X* (the infiltration score) determines two groups of observations regarding the response *Y* (the survival time and state), and these two groups have the largest statistic between each other.

### 2.8. Pseudotime Analysis

For scRNA-seq data, we used the FindMarkers function in the Seurat R package to obtain the high variable genes of five subtype CD8^+^ T cells with *p* value < 0.05. We used the STRING database (https://www.string-db.org/) to construct the PPI network and then used Cytoscape (v3.10.1) with the cytoHubba plug-in (MCC algorithm, top 10 nodes) to screen hub genes. Hub genes with survival differences were selected, and violin plots were drawn to show their expression differences between gender, grade, stage, age, diagnosis, disease types, and tissues.

To explore differential trajectory among five CD8^+^ T cell subtypes, we conducted pseudotime analysis of CD8^+^ T cells with the slingshot R package with default parameters after dimensionality reduction.

### 2.9. PPI Analysis

To identify key regulator genes of cytotoxic_CD8_T_cell_1 differentiation, we performed expression analysis between high and low groups of cytotoxic_CD8_T_cell_1 infiltration. Using *p* value < 0.05 and |log2FC| > 1, we totally identified 96 DEGs. With these genes, we built the PPI interaction network and then ranked these genes with MCC algorithms and the top 10 important genes were extracted as candidate genes. Survival analysis showed that SELL, CD79B, and RAMP2 significantly correlated with TCGA-STAD patient survival status.

### 2.10. Real-Time PCR

Forty-eight hours after transfection, cells from each group were collected and their mRNA was reverse-transcribed into cDNA following the instructions provided by the reverse transcription kit (Takara). For qRT-PCR, the real-time fluorescent PCR instrument LightCycle96 was utilized. GAPDH served as the internal reference gene. The relative expression levels were calculated using the 2^-*ΔΔ*Ct method. The primer sequences are listed in Supporting Information 3: Table S1.

### 2.11. CCK-8 Assay Procedure

Cells were seeded into 96-well plates at a concentration of 2∗10^4^ cells per well and subsequently cultured in an incubator maintained at 37°C with 5% CO_2_. At specific time points posttransfection—12, 24, 36, and 48 h—10 *μ*L of CCK-8 solution was added to each well. Following this addition, the cells were returned to the incubator for an additional 2 h of incubation. Using a microplate reader (Thermo), the optical density value at 450 nm was then measured for each well.

### 2.12. EdU Staining

To assess cell proliferation, an EdU (5-ethynyl-2⁣′-deoxyuridine) proliferation assay was conducted. Briefly, cells transfected with siRNA or siNC were plated in 24-well plates at a density of 5 × 10^4^ cells per well. The cells were treated with 10 nM docetaxel for 48 h. Subsequently, the cells were washed with PBS and incubated in serum-free DMEM containing 10 *μ*mol/L EdU (Beyotime, China) for 2 h. After fixation, the cells underwent EdU staining and DNA staining, adhering strictly to the manufacturer's instructions, to detect the number of cycling cells during the EdU treatment period. The cells were then imaged using fluorescence microscopy, and the number of proliferating cells was averaged to determine the labeling index.

### 2.13. Colony Formation Assay

The cells were seeded in six-well plates at a density of 2000 cells per well and then grown for 14 days with the indicated treatments. After being fixed for 15 min with 4% paraformaldehyde (Beyotime, Cat#: P0099-100 mL) at room temperature, the colonies were stained with 1% crystal violet (Solarbio, Cat#: G1063) for 15 min.

### 2.14. Statistical Analysis

We performed statistical analyses of genes differently expressed in different subsets, KEGG, GO, and GSEA analyses, by using the corresponding packages or default methods in the software. Random arrangement tests exhibited statistical pathway activity. In tumor and normal tissue samples, we used Student's test to assess the frequency of different cell types and used statistical analysis to examine the levels of expression. The log-rank test was used to test the significance of the KM curves. The Kruskal–Wallis test was used to determine dynamic changes in cell proportion and levels of gene expression at various stages of pathology. We used the Kruskal–Wallis test to determine dynamic changes in cell proportions and gene expression levels at different clinical features.

## 3. Results

### 3.1. scRNA-seq Data Download and Processing

We downloaded the scRNA-seq dataset GSE150290 from the GEO database (https://www.ncbi.nlm.nih.gov/geo/) and screened 23 GC tissue samples from it, totaling 123,302 cells. Supporting Information 4: Figure S1A shows the gene number, gene count, mitochondrial percentage, and hemoglobin (HB) percentage. The Pearson correlation coefficient between sequencing depth and the number of genes detected is 0.86 (*p* < 2.2e − 16) (Supporting Information 4: Figure S1B**)**, whereas the correlation coefficients between sequencing depth and both mitochondrial abundance (Supporting Information 4: Figure S1C) and HB concentration are −0.01 each (Supporting Information 4: Figure S1D). These data indicate a positive correlation between sequencing depth and the number of genes measured. In this study, for single-cell data, we have established the following screening criteria: the gene count primarily ranges from 300 to 7000, the gene expression level does not exceed 100,000, the proportion of mitochondrial content is less than 10%, and the proportion of HB content is less than 1%. Based on these, we selected 2000 highly expressed and DEGs for principal component analysis (PCA), and the results indicated that all 16 principal components were highly significant (Supporting Information 4: Figure S1E).

### 3.2. CD8^+^ T Cell Subtype Analysis

**Figure 1:**
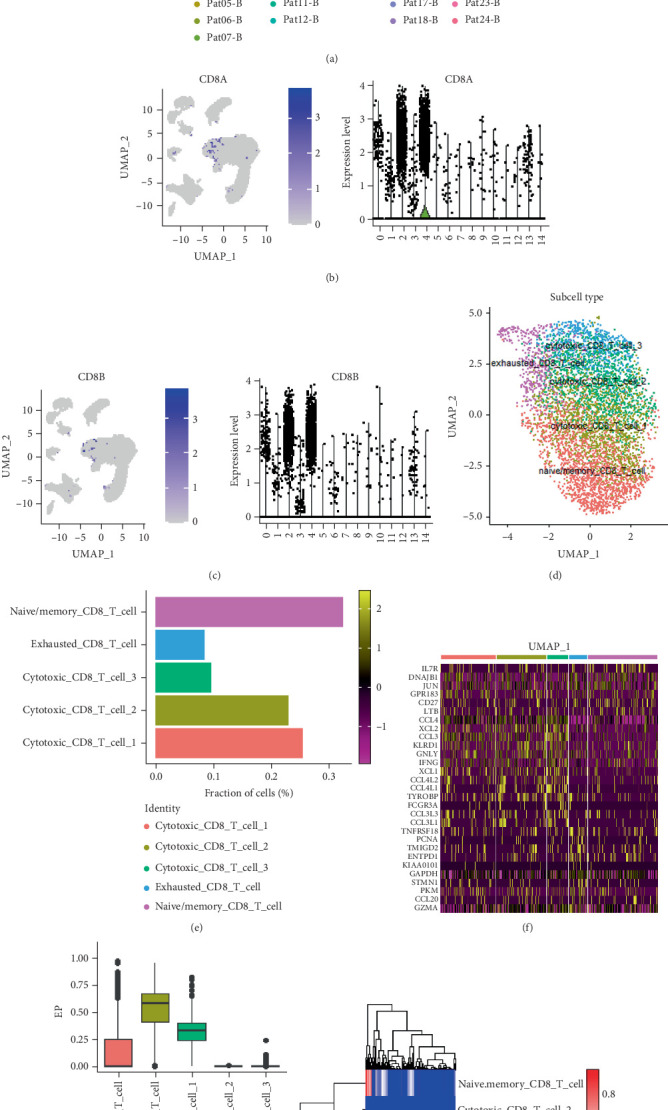
CD8^+^ T cell subtype analysis. (a) Reanalysis CD8^+^ T cells. (b, c) Expression of CD8A and CD8B in each CD8^+^ T cell cluster. (d) Annotated CD8^+^ T cell subtype. (e) Proportion of five CD8^+^ T subtypes in total CD8^+^ T cells. (f) Marker gene expression heatmap of the five subtypes. (g) Estimated infiltration level of the five subtypes in TCGA-STAD. (h) Heatmap showing infiltration levels of the five subtypes in TCGA-STAD.

To conduct an in-depth analysis of CD8^+^ T cells, we employed the UMAP clustering method and successfully identified 15 distinct cell clusters (Figure 1a). Of particular note is that Cluster 4 has become the primary residence for CD8A (Figure 1b) and CD8B (Figure 1c) cells, encompassing a total of 5380 cells. We selected Cluster 4 for further analysis and reapplied the UMAP clustering technique, ultimately successfully identifying five subsets of CD8^+^ T cells (Figure 1d).

These CD8^+^ T cell subsets exhibit their unique markers: naive/memory CD8^+^ T cells are characterized by the markers including TCF7, GPR183, CCR7, S100A10, IL7R, LEF1, SELL, SATB1, and LTB; cytotoxic CD8^+^ T cells are identified by the markers such as NKG7, PRF1, GZMK, and GZMA, while exhausted CD8^+^ T cells display a series of markers including GIMAP6, CXCL13, CXCR6, SPB1, IRF4, LAYN, HSPH1, CTLA4, LAG3, PDCD1 (also known as PD-1), TIGIT, and HAVCR2 (also known as TIM-3). Based on these distinctive markers, we have classified these five cell subsets as follows: naive/memory CD8^+^ T cells, exhausted CD8^+^ T cells, and three distinct cytotoxic CD8^+^ T cell subsets (labeled as cytotoxic CD8^+^ T Cells 1, 2, and 3) (Figure 1d).

In the single-cell dataset of GC, the proportion of cells within each subgroup showed that naive/memory CD8^+^ T cells accounted for the highest proportion, followed by cytotoxic CD8^+^ T Cells 1, while exhausted CD8^+^ T cells accounted for the lowest proportion (Figure 1e). Additionally, we conducted an analysis of the top 30 DEGs across the five CD8^+^ T cell subsets (Figure 1f). Utilizing the transcriptome data from TCGA-STAD, we calculated the proportions of various T cell subsets using GDC (Figure 1g) and CIBERSORTx (Figure 1h). The results indicated that the proportions of exhausted CD8^+^ T cells and cytotoxic CD8^+^ T Cell 1 were the highest.

### 3.3. Prognostic and Clinical Correlation Analysis

**Figure 2:**
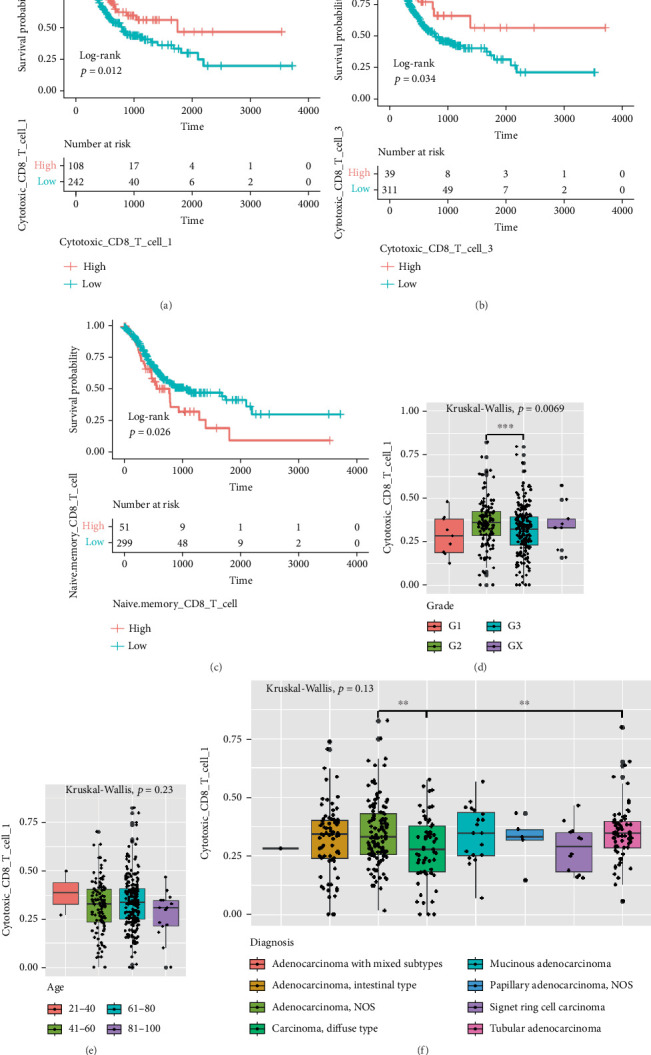
Prognostic and clinical correlation analysis. (a) The KM curves of cytotoxic CD8^+^ T Cell 1. (b) The KM curves of cytotoxic CD8^+^ T Cell 3. (c) The KM curves of naive/memory CD8^+^ T cells. (d) Infiltration levels of cytotoxic CD8^+^ T Cell 1 in different grades of TCGA-STAD. (e) Infiltration levels of cytotoxic CD8^+^ T Cell 1 at different age groups. Two samples for the 21–40 age group, 115 samples for the 41–60 age group, 215 samples for the 61–80 age group, and 15 samples for the 81–100 age group. (f) Infiltration levels of cytotoxic CD8^+^ T Cell 1 in different TCGA-STAD subtypes.

The results of survival analysis using the log-rank test showed significant differences in survival between groups with different characteristics, including high and low proportions of cytotoxic CD8^+^ T Cell Subset 1 (Figure 2a,b), as well as the proportion of naive/memory CD8^+^ T cells (Figure 2c). However, the survival difference observed among exhausted CD8^+^ T Cell 3 did not reach statistical significance (Supporting Information 5: Figure S2A). In addition, due to the extremely limited number of cytotoxic CD8^+^ T Cell 2, we cannot calculate the survival difference for them. The above results showed that cytotoxic CD8^+^ T1 cells had a high proportion and high tumor cell lysis ability, so this subpopulation was selected for further investigation.

To gain a deeper understanding of the characteristics of this subpopulation, we conducted a detailed analysis of its distribution under various conditions. In terms of gender distribution, the proportion of cytotoxic CD8^+^ T Cell 1 did not show significant differences (Supporting Information 5: Figure S2B). However, when we examined its distribution across different grade groups, we found significant differences (Figure 2d), with the highest ratio observed in G2-grade GC. Additionally, despite the different stages of GC, the proportion of cytotoxic CD8^+^ T Cell 1 remained relatively constant (Supporting Information 5: Figure S2C). Notably, the lowest proportion of cytotoxic CD8^+^ T Cell 1 was found in the 81–100 age group (Figure 2e). Meanwhile, we also observed that there were no significant differences in the proportion of cytotoxic CD8^+^ T Cell 1 among different diagnoses (Figure 2f), disease types (Supporting Information 5: Figure S2D), and tissues (Supporting Information 5: Figure S2E). Intriguingly, statistically significant differences emerged in the proportion of cytotoxic CD8^+^ T Cell 1 within diagnosis subgroups (Figure 2f).

### 3.4. Functional Enrichment Analysis and Pseudotime Analysis

**Figure 3:**
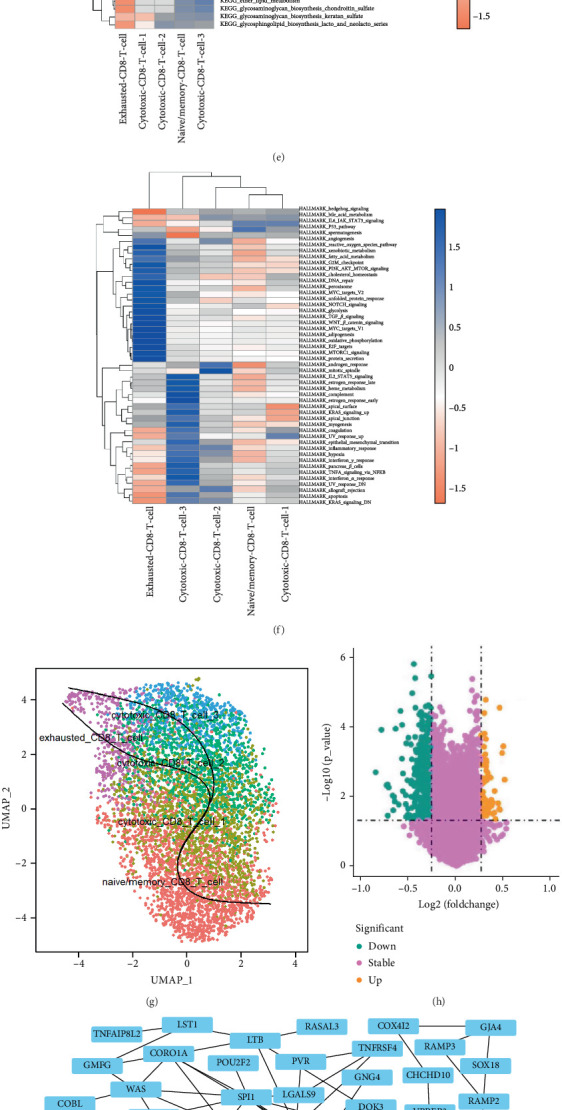
Functional enrichment analysis and pseudotime analysis. (a) Top 10 enriched biological process terms. (b) Top 10 enriched cellular component terms. (c) Top 10 enriched molecular function terms. (d) Top 10 enriched KEGG pathways. (e) Top 30 differentially enriched pathways between five CD8^+^ T cell subtypes. (f) Heatmap of enrichment score of each hallmark gene set in five CD8. (g) Differentiation trajectory of the five CD8^+^ T cell subtypes. (h) Volcano plot of the high and low cytotoxic CD8^+^ T Cell 1. (i) PPI network of the differentially expressed genes between high and low cytotoxic CD8^+^ T Cell 1. (j) Top 10 key gene's interaction network ranked by MCC (maximum clique centrality) algorithm. (k) Survival analysis between SELL high- and low-expression groups. (l) Survival analysis between CD79B high- and low-expression groups. (m) Survival analysis between RAMP2 high- and low-expression groups.

To explore the presence of heterogeneous pathways within CD8^+^ T cell subsets, we performed GO and KEGG analyses. The GO analysis revealed that cytotoxic CD8^+^ T Cell 1 exhibited distinct biological processes (Figure 3a), cellular components (Figure 3b), and molecular functions (Figure 3c). The KEGG results further indicated that these cytotoxic CD8^+^ T Cells 1 were associated with ubiquitin-mediated proteolysis (Figure 3d). To gain a deeper understanding of the heterogeneity among various subgroups, we also examined the metabolic pathway activity and hallmark immune checkpoint pathway activity within cell subgroups. Our analysis of metabolic pathway activity in cell subsets uncovered that exhaustive CD8^+^ T cells underwent the most significant alterations (Figure 3e). Moreover, the hallmark pathway activity analysis showed that both exhausted CD8^+^ T cells and cytotoxic CD8^+^ T Cells 3 exhibited the most altered pathway activity (Figure 3f).

Subsequently, we conducted a cell differentiation trajectory analysis. This analysis identified two lineages, both originating from naive/memory CD8^+^ T cells and progressing to exhausted CD8^+^ T cells, with distinct intermediate cytotoxic CD8^+^ T cell subgroups (Figure 3g). The results of the cell differentiation trajectory analysis were consistent with the actual biological process.

Building on the insights gained from the pathway activity and cell differentiation analyses, we next focus on the identification of prognostic and therapeutic markers. Utilizing the TCGA database, we initially screened out 422 DEGs based on significant survival differences, with 76 upregulated genes and 346 downregulated genes as the volcanic map showed (Figure 3h). After considering the fold change and *p* value, we screened out 95 DEGs. Based on these DEG data, we successfully constructed a PPI network (Figure 3i) and further identified key hub genes using the cytoHubba tool. Finally, we identified the top 10 key genes, which are SPI1, CORO1A, NCF1, SELL, WAS, LGALS9, TNFRSF4, CD79B, LTB, and RAMP2 (Figure 3j). Further analysis of the log-rank test revealed that, among these 10 core genes, the survival difference for SELL was statistically significant (Figure 3k), with CD79B (Figure 3l) and RAMP2 (Figure 3m) also demonstrating significant survival differences.

### 3.5. Identification of Prognostic and Therapeutic Markers

**Figure 4:**
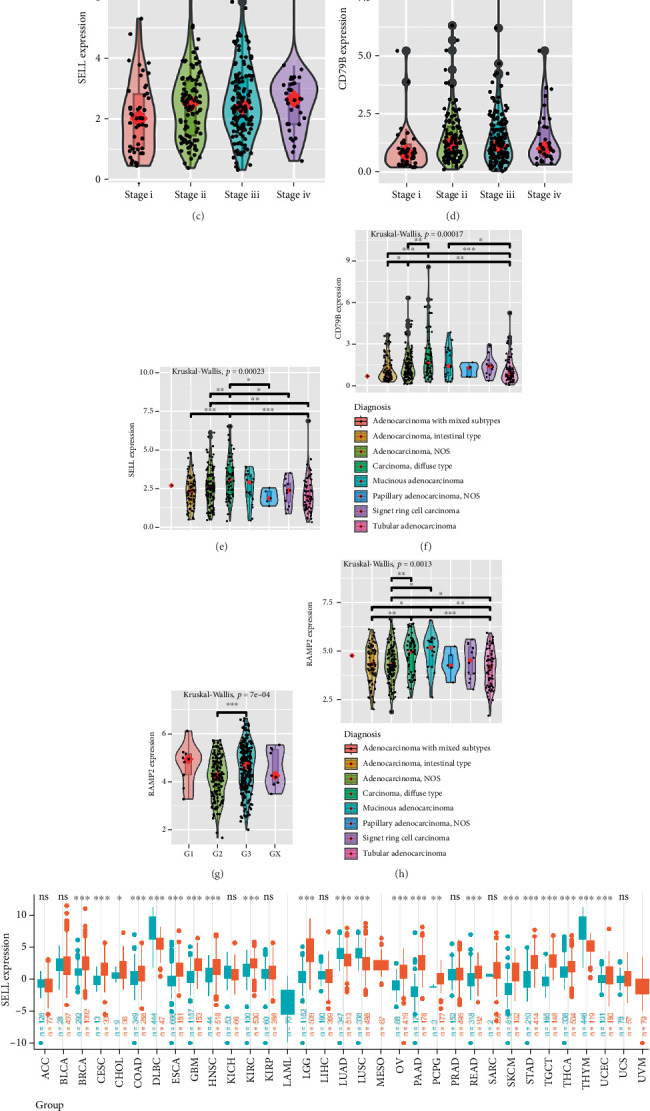
Identification of prognostic and therapeutic markers. (a) Expression level of SELL in different TCGA-STAD grades. (b) Expression level of CD79B in different TCGA-STAD grades. (c) Expression level of SELL in different TCGA-STAD stages. (d) Expression level of CD79B in different TCGA-STAD stages. (e) Expression level of SELL in different TCGA-STAD subtypes. (f) Expression level of CD79B in different TCGA-STAD subtypes. (g) Expression level of RAMP2 in different TCGA-STAD grades. (h) Expression level of RAMP2 in different TCGA-STAD subtypes. (i) Expression level of SELL between tumor and normal groups in various tumors. (j) Expression level of CD79B between tumor and normal groups in different tumors.

We investigated the expression levels of these genes across various clinical characteristics, which encompass gender, age, GC staging and grading, diagnostic types, specific types of GC, and tissues. The results indicate that the expression of SELL, CD79B, and RAMP2 genes exhibits significant differences in certain clinical characteristics. Specifically, the expression levels of SELL and CD79B show notable variations in GC grading (Figure 4a,b), staging (Figure 4c,d), and diagnostic types (Figure 4e,f). Although the RAMP2 gene does not demonstrate significant differences in GC staging (Supporting Information 6: Figure S3A), it does exhibit distinct expression differences in grading (particularly between G2 and G3) (Figure 4g) and diagnostic types (Figure 4h). However, it is noteworthy that no significant differences in the expression of SELL, CD79B, and RAMP2 were found across different dimensions such as gender (Supporting Information 6: Figure S3B–D), age (Supporting Information 6: Figure S3E–G), tissue location (Supporting Information 6: Figure S3H–J), and pathological types (Supporting Information 6: Figure S3K–M) (*p* > 0.05).

Ultimately, we conducted a thorough analysis of the expression patterns of these genes across various cancer types and uncovered that, in GC, the expression levels of SELL (Figure 4i) and RAMP2 (Figure 4j) are significantly elevated compared to normal tissue. Notably, although the expression level of CD79B did not exhibit significant fluctuations in comparative analyses (Supporting Information 6: Figure S3N), its importance in GC cannot be overlooked. This series of discoveries strongly suggests that SELL, CD79B, and RAMP2 have the potential to emerge as novel and highly promising prognostic indicators and therapeutic targets in the field of GC, opening up new perspectives and possibilities for precision medicine in this disease.

### 3.6. Experimental Validation of Hub Genes

**Figure 5:**
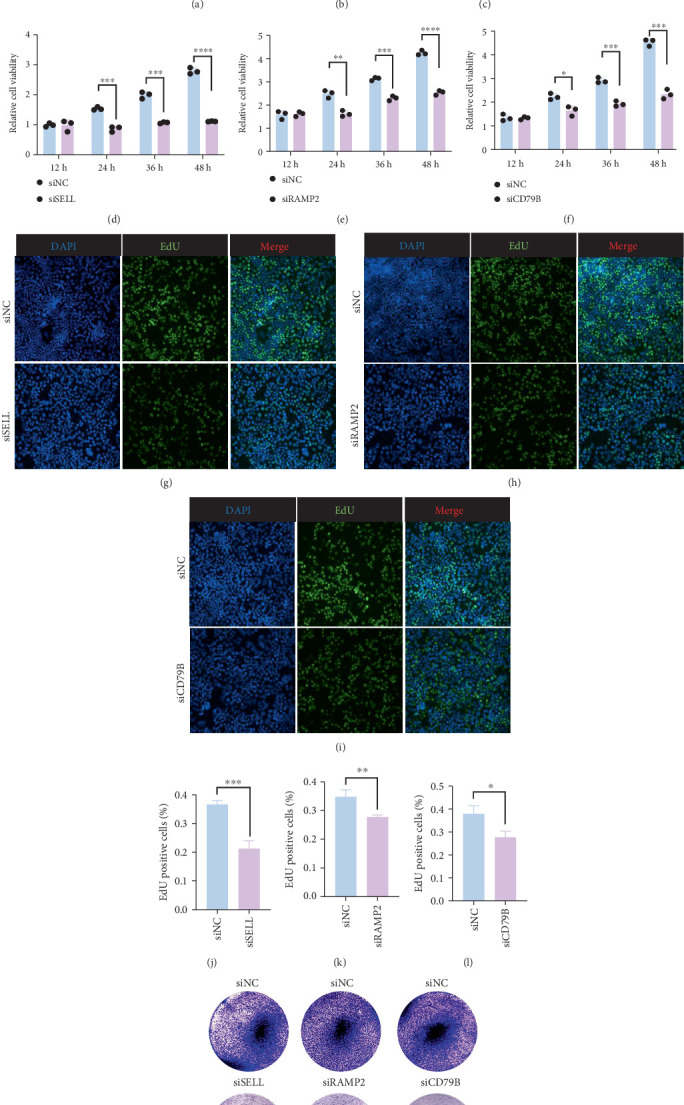
Experimental validation of hub genes. (a) Relative SELL expression in normal gastric epithelial cells (GES1) and GC cells (MKN28 and HCG27) analyzed by real-time PCR. (b) Relative RAMP2 expression in GES1 and GC cells (MKN28 and HCG27) analyzed by real-time PCR. (c) Relative CD79B expression in normal GES1 and gastric cancer cells (MKN28 and HCG27) analyzed by real-time PCR. (d) Results of the CCK-8 assay of MKN28 cells incubated with siSELL for the indicated time. (e) Results of the CCK-8 assay of MKN28 cells incubated with siRAMP2 for the indicated time. (f) Results of the CCK-8 assay of MKN28 cells incubated with siCD79B for the indicated time. (g, j) Results of the EdU assay of MKN28 cells incubated with siSELL. (h, k) Results of the EdU assay of MKN28 cells incubated with siRAMP2. (i, l) Results of the EdU assay of MKN28 cells incubated with siCD79B. (m) 2 × 10^3^ MKN28 cells were cultured in six-well plates per well for 24 h and then treated with the indicated siRNA and cultured in medium containing 10% FBS for 14 days. Colony image was taken.

To validate the expression of these three disease-related hub genes, we compared their expression in normal gastric epithelial cells and GC cells. The results showed the expression differences of SELL, RAMP2, and CD79B exhibited an upward trend in expression in GC cells with statistical significance (Figures 5a, 5b, and 5c). Furthermore, we used siRNA to knock down the expressions of SELL, RAMP2, and CD79B in GC cells. Cell viability (Figures 5d, 5e, and 5f) and proliferation (Figures 5g, 5h, 5i, 5j, and 5l) experiments demonstrated that the expression of these three genes was significantly correlated with the proliferation of GC cells. Besides, after treatment with siRNA, the number of clone-forming colonies was significantly reduced, confirming that knocking down the expression of SELL, RAMP2, and CD79B inhibited the proliferation of GC cells (Figure 5m).

### 3.7. SELL, RAMP2, and CD79B Coordinately Regulate Multiple Oncogenic Pathways in GC

**Figure 6:**
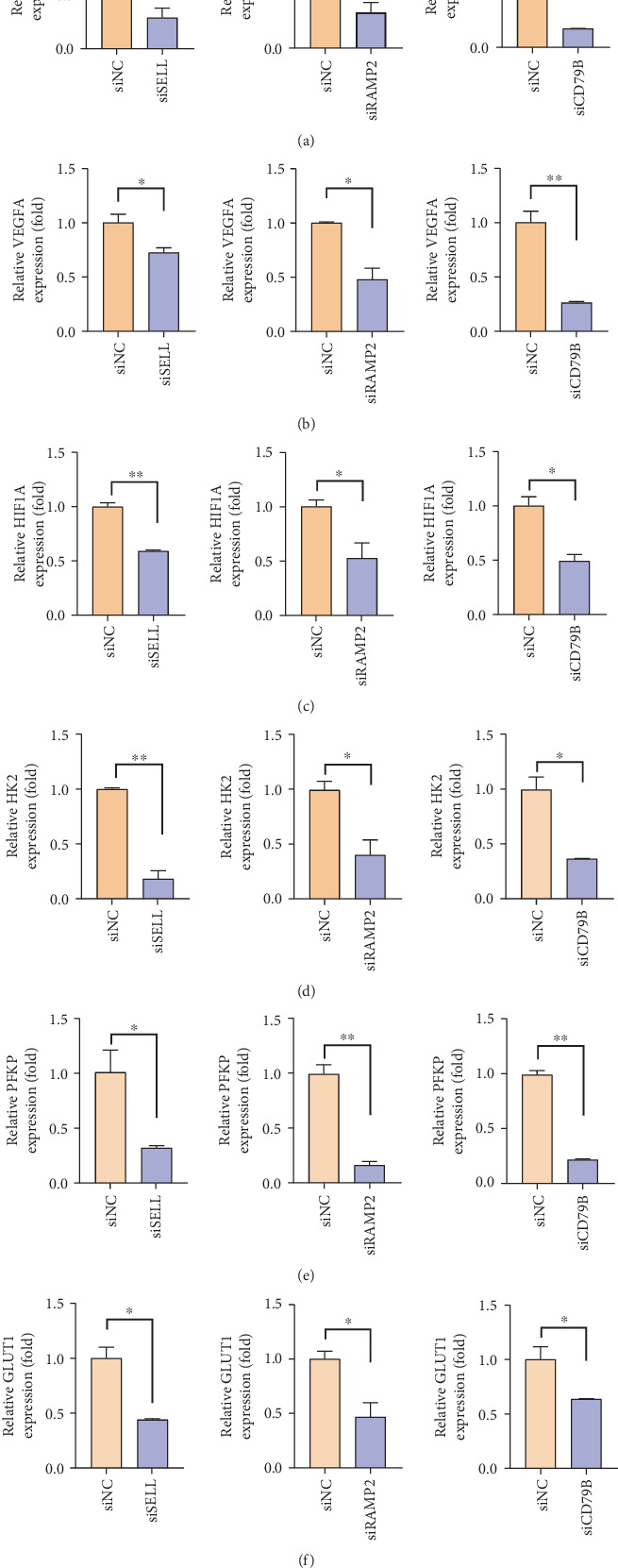
SELL, RAMP2, and CD79B coordinately regulate multiple oncogenic pathways in gastric cancer. (a) Relative MMP2 expression analyzed by real-time PCR. (b) Relative VEGFA expression analyzed by real-time PCR. (c) Relative HIF1A expression analyzed by real-time PCR. (d) Relative HK2 expression analyzed by real-time PCR. (e) Relative PFKP expression analyzed by real-time PCR. (f) Relative GLUT1 expression analyzed by real-time PCR.

To investigate the functional roles of SELL, RAMP2, and CD79B in GC progression, we performed siRNA-mediated knockdown of these genes in GC cell lines and assessed their impacts on tumor-associated pathways via qPCR. We found that knockdown of SELL, RAMP2, or CD79B significantly downregulated MMP2 (a key mediator of extracellular matrix degradation), VEGFA (a proangiogenic factor), and HIF1A (a hypoxia-inducible transcription factor) compared to scramble siRNA controls (Figures 6a, 6b, and 6c). As indicated in Figure 3, alterations in metabolic pathways may contribute to cellular heterogeneity, and given the central role of glycolysis—a highly scrutinized metabolic process—we specifically examined three critical glycolytic genes (HK2, PFKP, and GLUT1). Following siRNA-mediated knockdown of SELL, RAMP2, and CD79B in GC cells, qPCR analysis revealed significant downregulation of the aforementioned glycolysis pathway components HK2 (Figure 6d), PFKP (Figure 6e), and GLUT1 (Figure 6f). These findings demonstrate that SELL, RAMP2, and CD79B collaboratively drive GC progression by regulating invasion, angiogenesis, hypoxia adaptation, and metabolic reprogramming. Their targeted intervention may provide novel strategies to suppress multidimensional malignant phenotypes in GC.

## 4. Discussion

Immunotherapy has emerged as a cornerstone of cancer treatment, yet its application in GC remains hindered by an incomplete understanding of TME dynamics [20–22]. A comprehensive understanding of the internal immune microenvironment of cancer tissue is crucial for developing novel immunotherapies. Notably, CD8^+^ T cells, the cornerstone effector T cells in current tumor immunotherapy [23], recognize tumor-associated antigens presented on cancer cell surfaces as major histocompatibility complex class I molecules [24].

Single-cell sequencing technology is an effective tool for studying the immune microenvironment of GC, playing a vital role in immune cell therapy and antibody drug development [25, 26]. This research provides a deeper understanding of the mechanism and potential targets for the application of immunotherapy in the field of GC. Through scRNA-seq, we classified CD8^+^ T cells into five subsets, including three cytotoxic subpopulations with divergent prognostic impacts. Cytotoxic CD8^+^ T1 cells—enriched in G2-grade tumors and associated with improved survival—represent a therapeutically actionable subset, underscoring the importance of subset-specific functional states in clinical outcomes. Our in vitro experiments revealed that SELL, CD79B, and RAMP2 are functionally critical for GC cell proliferation. SELL (a regulator of lymphocyte homing) [27–29], RAMP2 (implicated in vascular remodeling) [30, 31], and CD79B (a B cell receptor component) [32, 33] exhibited tumor-specific upregulation and functional roles in GC proliferation, as validated by siRNA knockdown. These genes correlate with tumor grade and stage, suggesting utility as biomarkers for patient stratification. Potential mechanisms of these genes may include L-selectin-mediated lymphocyte infiltration (SELL), AMPK-dependent glycolysis (RAMP2), and BCR signaling feedback (CD79B). Our pseudotime analysis revealed differentiation trajectories from naïve to exhausted CD8^+^ T cells, accompanied by metabolic reprogramming and immune checkpoint upregulation (e.g., PDCD1 and CTLA4). These findings align with prior studies in NSCLC and liver cancer [15, 16] yet uniquely implicate ubiquitin-mediated proteolysis in cytotoxic subset dysfunction—a potential target for reinvigorating antitumor immunity.

Our findings pave the way for personalized immunotherapy in GC through three actionable strategies: (1) biomarker-driven stratification—the SELL/RAMP2 expression signature could identify patients likely to benefit from PD-1/CTLA-4 inhibitors or antiangiogenic agents, while elevated CD79B levels may predict responsiveness to B cell-targeted therapies (e.g., rituximab analogs); (2) targeted combination strategies—combined inhibition of RAMP2 and VEGF pathways could disrupt tumor vasculature while enhancing T cell infiltration, and small-molecule SELL inhibitors might synergize with checkpoint blockade to suppress metastasis; (3) dynamic monitoring—longitudinal profiling of these genes during therapy could enable adaptive treatment adjustments to circumvent resistance. Different from conventional approaches relying on bulk transcriptome profiles or generic immune scoring systems, our three-gene signature derived from single-cell sequencing synthesizes three important dimensions: cellular subset specificity, prognostic significance, and therapeutic tractability. This new framework provides a more adaptable instrument for clinical management, particularly in directing immunotherapy and combination therapy for gastric carcinoma. Together, these approaches exemplify a precision oncology framework to optimize immunotherapy outcomes in GC.

## 5. Conclusions

In conclusion, this study establishes CD8^+^ T cell heterogeneity as a central determinant of GC progression and therapy resistance. By integrating single-cell analytics with functional genomics, we propose a precision medicine framework to tailor immunotherapies based on SELL/CD79B/RAMP2 profiles—a strategy poised to transform GC management.

## 6. Limitations

Although our in vitro experiments have confirmed the roles of SELL, CD79B, and RAMP2, animal-based experiments are also essential for the improvement of clinical transformation. We plan to use GC mouse models to assess the therapeutic relevance of these genes in future studies, particularly in relation to immunotherapy. This will help strengthen the clinical translational value of our findings.

## Data Availability

The data that support the findings of this study are available from the corresponding authors upon reasonable request.

## Supporting Information

Supporting Information 1 The transcriptome data of TCGA-STAD.

Supporting Information 2 The TCGA clinical cohort data.

Supporting Information 3 Table S1: Primer sequences used for PCR.

Supporting Information 4 Figure S1: Quality control chart of the single-cell data set. (A) The number of detected genes, sequence depth, mitochondria (mt) percentage, and HB percentage. (B) Scatter plot between sequence depth and number of detected genes. (C) Scatter plot between sequence depth and mitochondria percentage. (D) Scatter plot between sequence depth and HB percentage. (E) Elbow plot for the top 20 PCs.

Supporting Information 5 Figure S2: Prognostic and clinical correlation analysis. (A) Survival analysis between exhausted CD8^+^ T cell infiltration high and low groups. (B) Infiltration level of cytotoxic CD8^+^ T cells between male and female. (C) Infiltration level of cytotoxic CD8^+^ T Cell 1 among Stages I–IV. (D) Infiltration level of cytotoxic CD8^+^ T Cell 1 between TCGA-STAD subtypes. (E) Infiltration level of cytotoxic CD8^+^ T Cell 1 among different tissues of origin.

Supporting Information 6 Figure S3: Identification of prognostic and therapeutic markers. (A) Expression of RAMP2 in Stages I–IV. (B) Expression of SELL between male and female. (C) Expression of CD79B between male and female. (D) Expression of RAMP2 between male and female. (E) Expression of SELL in different periods of time. (F) Expression of RAMP2 in different periods of time. (G) Expression of RAMP2 in different periods of time. (H) Expression of SELL in different tissues of origin. (I) Expression of CD79B in different tissues of origin. (J) Expression of RAMP2 in different tissues of origin. (K) Expression of SELL in different TCGA-STAD subtypes. (L) Expression of CD79B in different TCGA-STAD subtypes. (M) Expression of RAMP2 in different TCGA-STAD subtypes. (N) Expression of RAMP2 in different TCGA-STAD subtypes.
