# Supplementary material for: A CD8+ T Cell Infiltration–Driven Prognostic Signature for Gastric Cancer: Bridging Tumor Immunity and Clinical Outcomes
Source: Int J Genomics. 2025 Jun 13;2025:6629479. doi: 10.1155/ijog/6629479 (PMC12181657; doi:10.1155/ijog/6629479)
Supplement: Supporting Information 3 — Table S1: Primer sequences used for PCR. [file 6629479.f3.docx]

**Supplementary Table 1**

Table S1. Primer sequences used for PCR.

| **Gene** | **Sequence (5’→3’)** |
| --- | --- |
| Human *sell* forward | 5’- TGCCGAGACAATTACACAGATTT -3’ |
| Human *sell* reverse | 5’- TGAAAGGCAGAGTCTTCTCCAG -3’ |
| Human *ramp2* forward | 5’- TCCTATCGAAAAGGATTGGTGC -3’ |
| Human *ramp2* reverse | 5’- GTTGGCAAAGTGGATCTGGTG -3’ |
| Human *cd79b* forward | 5’- AGGGCCTGGACATTGACCA -3’ |
| Human *cd79b* reverse | 5’- CACCTACAGACCACTTCACTTC -3’ |
| Human *vegfa* forward | 5’-AGGGCAGAATCATCACGAAGT -3’ |
| Human *vegfa* reverse | 5’- AGGGTCTCGATTGGATGGCA -3’ |
| Human hif1α forward | 5’-GAACGTCGAAAAGAAAAGTCTCG -3’ |
| Human hif1α reverse | 5’-CCTTATCAAGATGCGAACTCACA -3’ |
| Human *hk2* forward | 5’-TTGACCAGGAGATTGACATGGG -3’ |
| Human *hk2* reverse | 5’-CAACCGCATCAGGACCTCA -3’ |
| Human *pfkp* forward | 5’-GACCTTCGTTCTGGAGGTGAT -3’ |
| Human *pfkp* reverse | 5’-CACGGTTCTCCGAGAGTTTG-3’ |
| Human *glut1* forward | 5’-ATTGGCTCCGGTATCGTCAAC -3’ |
| Human *glut1* reverse | 5’-GCTCAGATAGGACATCCAGGGTA -3’ |
