# Supplementary material for: A CD8+ T Cell Infiltration–Driven Prognostic Signature for Gastric Cancer: Bridging Tumor Immunity and Clinical Outcomes
Source: Int J Genomics. 2025 Jun 13;2025:6629479. doi: 10.1155/ijog/6629479 (PMC12181657; doi:10.1155/ijog/6629479)
Supplement: Supporting Information 4 — Figure S1: Quality control chart of the single-cell data set. (A) The number of detected genes, sequence depth, mitochondria (mt) percentage, and HB percentage. (B) Scatter plot between sequence depth and number of detected genes. (C) Scatter plot between sequence depth and mitochondria percentage. (D) Scatter plot between sequence depth and HB percentage. (E) Elbow plot for the top 20 PCs. [file 6629479.f4.docx]

**
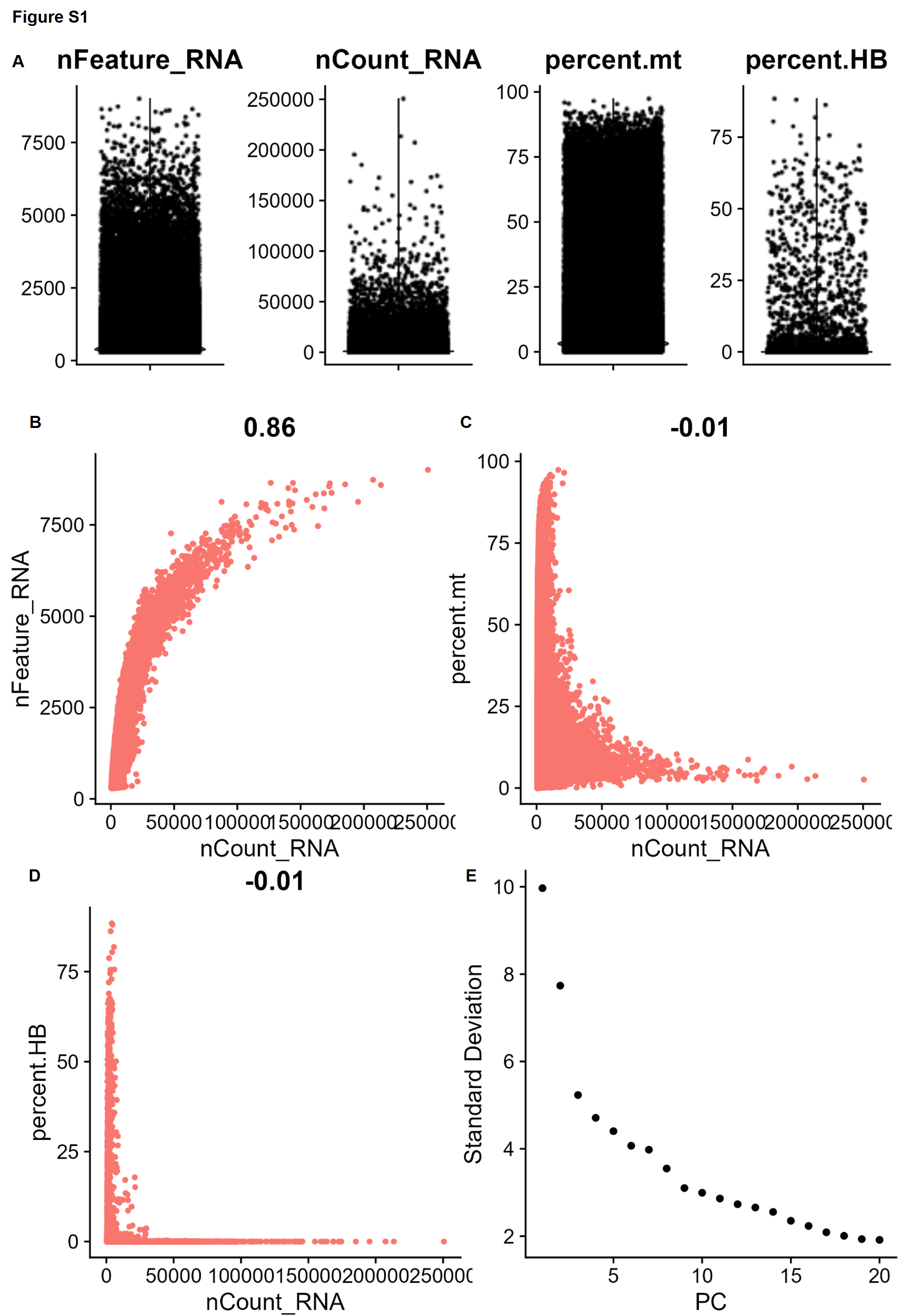
**

**Figure S1.**

Quality control chart of the single-cell data set. (A) The number of detected genes, sequence depth, mitochondria (mt) percentage and HB percentage. (B) Scatter plot between sequence depth and number of detected genes. (C) Scatter plot between sequence depth and mitochondria percentage. (D) Scatter plot between sequence depth and HB percentage. (E) Elbow plot for the top 20 PCs.
