# Supplementary material for: A CD8+ T Cell Infiltration–Driven Prognostic Signature for Gastric Cancer: Bridging Tumor Immunity and Clinical Outcomes
Source: Int J Genomics. 2025 Jun 13;2025:6629479. doi: 10.1155/ijog/6629479 (PMC12181657; doi:10.1155/ijog/6629479)
Supplement: Supporting Information 5 — Figure S2: Prognostic and clinical correlation analysis. (A) Survival analysis between exhausted CD8+ T cell infiltration high and low groups. (B) Infiltration level of cytotoxic CD8+ T cells between male and female. (C) Infiltration level of cytotoxic CD8+ T Cell 1 among Stages I–IV. (D) Infiltration level of cytotoxic CD8+ T Cell 1 between TCGA-STAD subtypes. (E) Infiltration level of cytotoxic CD8+ T Cell 1 among different tissues of origin. [file 6629479.f5.docx]

**
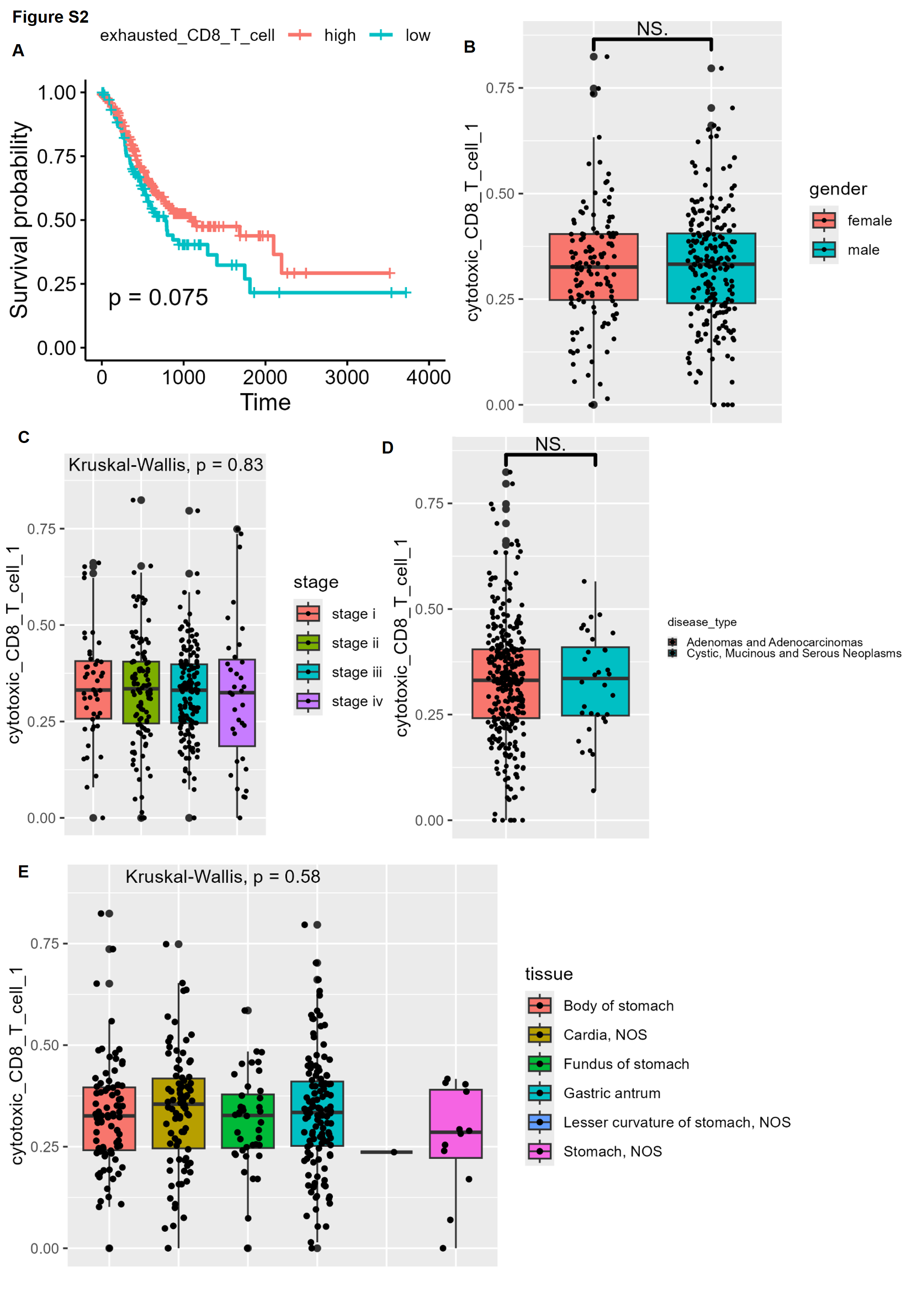
**

**Figure S2.**

Prognostic and clinical correlation analysis. (A) Survival analysis between exhausted CD8^+^T cell infiltration high and low group. (B) Infiltration level of cytotoxic CD8^+^T cell between male and female. (C) Infiltration level of cytotoxic CD8^+^T cell 1 among stage I to IV. (D) Infiltration level of cytotoxic CD8^+^T cell 1 between TCGA-STAD subtype. (E) Infiltration level of cytotoxic CD8^+^T cell 1 among different tissue of origin.
