# Supplementary material for: A CD8+ T Cell Infiltration–Driven Prognostic Signature for Gastric Cancer: Bridging Tumor Immunity and Clinical Outcomes
Source: Int J Genomics. 2025 Jun 13;2025:6629479. doi: 10.1155/ijog/6629479 (PMC12181657; doi:10.1155/ijog/6629479)
Supplement: Supporting Information 6 — Figure S3: Identification of prognostic and therapeutic markers. (A) Expression of RAMP2 in Stages I–IV. (B) Expression of SELL between male and female. (C) Expression of CD79B between male and female. (D) Expression of RAMP2 between male and female. (E) Expression of SELL in different periods of time. (F) Expression of RAMP2 in different periods of time. (G) Expression of RAMP2 in different periods of time. (H) Expression of SELL in different tissues of origin. (I) Expression of CD79B in different tissues of origin. (J) Expression of RAMP2 in different tissues of origin. (K) Expression of SELL in different TCGA-STAD subtypes. (L) Expression of CD79B in different TCGA-STAD subtypes. (M) Expression of RAMP2 in different TCGA-STAD subtypes. (N) Expression of RAMP2 in different TCGA-STAD subtypes. [file 6629479.f6.docx]

**
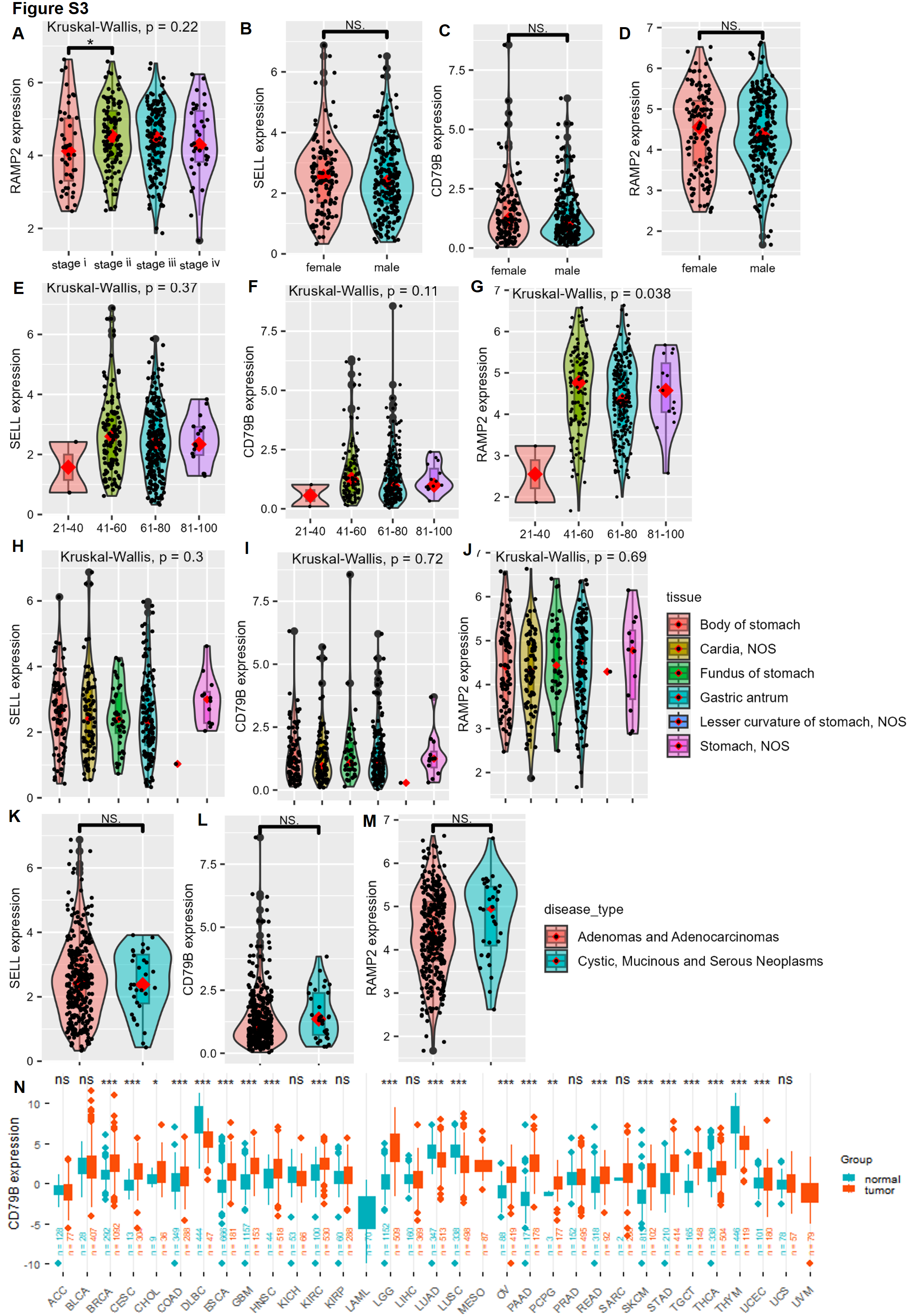
**

**Figure S3.**

Identification of prognostic and therapeutic markers. (A) Expression of RAMP2 in stage I to IV. (B) Expression of SELL between male and female. (C) Expression of CD79B between male and female. (D) Expression of RAMP2 between male and female. (E) Expression of SELL in different periods of time. (F) Expression of in different periods of time. (G) Expression of RAMP2 in different periods of time. (H) Expression of SELL in different tissue of origin. (I) Expression of CD79B in different tissue of origin. (J) Expression of RAMP2 in different tissue of origin. (K) Expression of SELL in different TCGA-STAD subtype. (L) Expression of CD79B in in different TCGA-STAD subtype. (M) Expression of RAMP2 in different TCGA-STAD subtype. (N) Expression of RAMP2 in different TCGA-STAD subtype.
